# Different Training Modalities Improve Energy Cost and Performance in Master Runners

**DOI:** 10.3389/fphys.2018.00021

**Published:** 2018-01-24

**Authors:** Lorenzo Pugliese, Simone Porcelli, Alessandra Vezzoli, Antonio La Torre, Fabio R. Serpiello, Gaspare Pavei, Mauro Marzorati

**Affiliations:** ^1^Institute of Molecular Bioimaging and Physiology, National Research Council, Segrate, Italy; ^2^Department of Biomedical Sciences for Health, Università degli Studi di Milano, Milan, Italy; ^3^Institute of Sport, Exercise and Active Living, College of Sport and Exercise Science, Victoria University, Melbourne, VIC, Australia; ^4^Department of Pathophysiology and Transplantation, Università degli Studi di Milano, Milan, Italy

**Keywords:** discontinuous high-intensity training, continuous moderate-intensity training, energy cost, performance, endurance athletes

## Abstract

**Purpose:** The aim of this study was to compare the effects of continuous moderate-intensity and discontinuous high-intensity training on running performance in master runners.

**Methods:** Thirty-four male master runners (47.2 ± 7.4 years) were assigned to three different groups: continuous moderate-intensity training (CMIT), discontinuous high-intensity training (DHIT), and control group (CON). CMIT and DHIT performed 8-week of supervised training (3 session·wk^−1^; ~35 km·wk^−1^) while CON maintained their normal training habits (3–4 session·wk^−1^; ~50 km·wk^−1^). Peak oxygen consumption ($\dot{V}$O_2peak_) and peak running speed (v_peak_) during incremental treadmill exercise, gas exchange threshold (GET), speed at GET, energy cost of running (Cr), and 5-km performance were evaluated before and after training.

**Results:** Following the training period, both CMIT and DHIT significantly reduced Cr (−4.4 and −4.9%, respectively, *P* < 0.05), increased speed at GET (3.4 and 5.7%, *P* < 0.05) and improved 5-km time-trial performance (3.1 and 2.2%, *P* < 0.05) whereas no differences were found for $\dot{V}$O_2peak_ and GET (as %$\dot{V}$O_2peak_). After training, v_peak_ improved only for DHIT (6%, *P* < 0.05). No differences were found in any variable for CON.

**Conclusions:** This study indicates that both CMIT and DHIT may positively affect running performance in middle-aged master runners. This improvement was achieved despite a significant reduction of the amount of weekly training volume.

## Introduction

Master athletes are typically defined as men and women older than 35 years who continue physical training throughout life and compete in organized events. The number of masters athletes involved in sport activities is continuously increasing, particularly in long-distance running events (Tanaka and Seals, 2008). For instance, statistics from United States indicate that marathon finishers aged over 40 years were 37,180 (26% of total finishers) in 1980, and 249,410 (49% of total finishers) in 2015 (Running USA Annual Marathon Report, 2015)^1^

Master athletes are still capable of accomplishing outstanding performances (Trappe et al., 2013). However, exercise performance inevitably declines with aging despite regular training and participation in sporting competitions (Donato et al., 2003; Reaburn and Dascombe, 2008; Tanaka and Seals, 2008; Brisswalter and Nosaka, 2013). $\dot{V}$O_2max_ seems to be the major determinant of this decrease whereas running economy and lactate threshold, when expressed relative to the percentage of $\dot{V}$O_2max_, appear well preserved with aging (Reaburn and Dascombe, 2008; Tanaka and Seals, 2008; Brisswalter and Nosaka, 2013).

In endurance master athletes, age-associated reduction in $\dot{V}$O_2max_ is due to a decline in both maximal cardiac output and maximal arterio-venous oxygen difference and it is likely mediated by a reduction in the exercise training “stimulus” (i.e., exercise-training intensity, session duration, and weekly frequency). Indeed, master athletes have often less time for training than young athletes do and the increased job- and family-related responsibilities may impinge on the availability of time and energy for the intensive training required to remain competitive (Tanaka and Seals, 2008). In addition, training stimulus can be inadequate because few master athletes still have coaches or follow structured training programs. The increased prevalence of exercise training-associated injuries in this population probably also contributes to their reduced training intensity and volume (Reaburn and Dascombe, 2008).

In general, the main modalities of training used to improve endurance exercise performance are: (i) continuous training at low- to moderate-intensity (CMIT) characterized by high volumes of training (> 30 min per session) with intensities below the “anaerobic threshold” or between 60 and 80% of $\dot{V}$O_2max_ (Kubukeli et al., 2002; Laursen and Jenkins, 2002; Seiler and Kjerland, 2006) and (ii) discontinuous high-intensity training (DHIT) characterized by repeated short bouts of exercise performed at an intensity corresponding to $\dot{V}$O_2max_ or above, separated by brief periods of low-intensity work or inactivity which allow a partial but often not a full recovery of resting heart rate and $\dot{V}$O_2_ values (Kubukeli et al., 2002; Laursen and Jenkins, 2002; Gibala et al., 2006). Until a few years ago, it was widely believed that DHIT was a prerogative of elite athletes or recreational runners accustomed to sustain strenuous efforts and exercises of low/moderate intensity and high-volume were primarily prescribed to sedentary or moderately trained subjects, as this was considered safer and effective to improve aerobic metabolism (Holloszy and Coyle, 1984; Laursen, 2010). However, several studies have shown that in adult sedentary or moderately trained subjects, DHIT might be an efficient strategy to improve running performance (Franch et al., 1998; Billat et al., 1999; Helgerud et al., 2007; Laursen, 2010). Indeed, an improved maximal oxygen uptake ($\dot{V}$O_2max_) (Milanović et al., 2015) and a reduced energy expenditure at submaximal running speeds (Franch et al., 1998; Billat et al., 1999; Helgerud et al., 2007; Iaia et al., 2009) have been reported and it is widely accepted that these both factors, in addition to the ability to sustain a high percentage of $\dot{V}$O_2max_ for an extended period of time (*f* $\dot{V}$O_2_), are the main physiological determinants of running performance (di Prampero, 1986; Capelli, 1999).

To the best of our knowledge, there is a lack of data on the effects of DHIT programs in middle-aged master runners with several years of training experience. Thus, the aim of this study was to compare the effects of CMIT and DHIT programs, characterized by the same volume, on running performance and the main physiological variables related to endurance performance in master runners. A group of runners, who maintained their training habits, was utilized as control. Our hypothesis was that DHIT, by affecting $\dot{V}$O_2max_ and energy cost of running, would be as effective as CMIT in ameliorating running performance.

## Materials and methods

### Participants

Thirty-four male master runners (age: 47.2 ± 7.4 years, height: 1.75 ± 0.06 m, body mass: 70.0 ± 8.8 kg, body mass index: 23.0 ± 1.9 kg·m^−2^) participated in the study. Athletes had a training experience of 15 ± 4 years and in the last 6 months they reported an average training volume of ~50 km·wk^−1^ with a training frequency of 3–4 sessions per week. Participants were not involved in a structured training program and they had independently managed their own training for at least 5 years. All of them competed at regional level on distances from 10 km to marathon. Before the start of the study, participants underwent a complete medical screening (medical history, physical examination, and ECG) to ensure that there were no contraindications to study participation. Each athlete was fully informed about the aims, methods and risks associated with participation and gave his written informed consent before the start of the study. All procedures were in accordance with the Declaration of Helsinki and the study was approved by the local Ethics Committee (IBFM-CNR-2013).

### Study design

Following the preliminary evaluation, participants were randomly assigned to three different groups: continuous moderate-intensity training (CMIT, *n* = 11); discontinuous high-intensity training (DHIT, *n* = 11) and control group (CON, *n* = 12). All participants were tested before (PRE) and after 8-week of training (POST). Thirty participants successfully completed the study (CMIT, *n* = 10; DHIT, *n* = 10; and CON, *n* = 10). Four subjects dropped out of the study: one subject for knee pain (CMIT group), and three for muscle injury occurred during training (one in DHIT and two in CON). Data obtained from these subjects were excluded from analysis.

### Tests and procedures

Participants were instructed to arrive at the laboratory in a rested and euhydrated state, about 3 h postprandial, and to avoid strenuous exercise in the 24 h preceding each testing session. In addition, they were invited to avoid alcohol and caffeine products intake 48 h before the exercise test. All laboratory exercise testing sessions were carried out in a well-ventilated laboratory at 19–21°C on a motorized treadmill (Laufergotest, Jaeger, Germany) set at 1% gradient to compensate for air resistance cost (Jones and Doust, 1996). The speed was frequently checked by belt length and count of rotations while subjects were running. On the first day, subjects performed a ramp incremental exercise test (IE) for determination of $\dot{V}$O_2peak_ and gas exchange threshold (GET). The protocol began with subjects running at 8 km·h^−1^ for 6 min and the belt speed was thereafter increased by 1 km·h^−1^ every minute until volitional exhaustion. The peak values of the main cardiovascular, respiratory, and metabolic parameters were taken as the highest 30-s mean value attained prior to the subject's volitional exhaustion. The GET was determined as described previously (Beaver et al., 1986) and speed at GET was the speed equivalent at that $\dot{V}$O_2_ value. Maximal speed (v_peak_) reached at the end of IE was also recorded.

At least 48 h following IE, subjects performed two repetitions of 6-min moderate intensity (at the speed equivalent to 60% of $\dot{V}$O_2peak_) constant load exercise (CLE) exercise, separated by at least a 20 min recovery period. Breath-by-breath data obtained in the two repetitions were pooled and time-aligned for each subject. Average steady-state values of the main cardiovascular, respiratory and metabolic parameters were calculated during the last 40–60 s of both trials. The energy cost of running (Cr, J·kg^−1^·m^−1^) was calculated during CLE as the ratio between net $\dot{V}$O_2_ (steady state $\dot{V}$O_2_-$\dot{V}$O_2_ measured at rest in standing position, mL·kg^−1^·m^−1^) and the progression speed; the unit conversion from mL O_2_ to metabolic J was achieved by considering the mean respiratory exchange ratio (RER) value (Margaria et al., 1963). As previously reported (Margaria et al., 1963), Cr is speed independent, at least for speeds wherein the air resistance is negligible (approximately up to 18 km·h^−1^).

Finally, participants completed a 5-km time trial on a 400-m outdoor track. PRE and POST running test was performed twice, at least 5 days apart, and the best performance time recorded. Weather conditions (temperature, relative humidity, absence of wind) were consistent between the trials. During trials, subjects received only information regarding the number of laps remaining. Performance time was measured using a manual stopwatch.

### Measurements

Pulmonary ventilation ($\dot{V}$E, in BTPS), O_2_ consumption ($\dot{V}$O_2_), and CO_2_ output ($\dot{V}$CO_2_), both in STPD, were determined breath-by-breath by a metabolic cart (Vmax29c; SensorMedics, Bilthoven, The Netherlands). Expiratory flow was determined by a mass flow sensor (hot wire anemometer), calibrated before each experiment by a 3 liters syringe at three different flow rates. $\dot{V}$O_2_ and $\dot{V}$CO_2_ were determined by continuously monitoring PO_2_ and PCO_2_ at the mouth throughout the respiratory cycle and from established mass balance equations. RER was calculated as $\dot{V}$CO_2_/$\dot{V}$O_2_. Calibration of O_2_ and CO_2_ analyzers was performed before each experiment by utilizing gas mixtures of known composition. Heart rate (HR) was determined from the ECG signal continuously monitored throughout the tests. At rest and at various times (1, 3, 5, and 7 min) during recovery, 20 μL of capillary blood was obtained from a preheated earlobe for the determination of blood lactate concentration ([La^−^]_b_) by an enzymatic method (Biosen C-Line; EKF, Germany).

### Training intervention

CMIT and DHIT groups trained 3 times per week during an 8-week period on a 400-m outdoor track. Three different types of training sessions were scheduled for each group, based on the individual speed at GET value. Total distance achieved during each session was calculated in order to obtain an identical training volume. For CMIT, sessions were: (i) 64.5 min at 70% speed at GET, (ii) 58.5 min at 80% speed at GET, and (iii) 54 min at 90% speed at GET. For DHIT, sessions were: (i) 18 × (1 min at 120% speed at GET followed by 2 min at 65% speed at GET), (ii) 18 × (1 min at 130% speed at GET followed by 2 min at 65% speed at GET), and (iii) 18 × (1 min at 140% speed at GET followed by 2 min at 65% speed at GET). All athletes in CMIT and DHIT groups received a detailed training plan before the start of the study and they trained under the supervision of a professional coach. As for CON, participants maintained their training habits during the 8-week period, consisting of 3/4 training session per week and about 50 km·week^−1^. No specific indications about training volume and intensity were given to these subjects but HR, speed, and total distance of each session were recorded. Competitions were not allowed during the entire period of the study for all groups.

### Statistical analysis

Results are presented as box and whiskers plot. The box extends from the 25th percentile to the 75th percentile, with a bold line at the median value. In the text and tables, results are expressed as mean ± SD and 95% CI of the differences is also reported. Data were analyzed using a two-way ANOVA for repeated measures (groups × time), excluding training volume data which was analyzed using a one-way ANOVA. *Post-hoc* analysis was completed using Bonferroni multiple comparisons. When significant effects of time were found, a paired Student's *t*-test was used to determine differences between PRE and POST. The relevance of the difference POST vs. PRE and its practical importance was defined using effect size (Batterham and Hopkins, 2006). A stepwise multiple regression analysis of the PRE data was also performed in order to extract a set of physiological variables which provided the optimal prediction of 5-km time trial. The level of significance was set at *P* < 0.05 (Prism 6.0, GraphPad Software, USA).

## Results

### Training volume

During the 8 weeks, training volume was significantly lower (*P* = 0.007) in both CMIT (34.1 ± 3.1 km·wk^−1^) and DHIT (33.3 ± 2.8 km·wk^−1^) compared to CON (51.8 ± 13.4 km·wk^−1^).

### Incremental exercise

Mean values of the main cardiovascular, respiratory, and metabolic variables obtained at PRE and POST in CMIT, DHIT, and CON are shown in Table 1. All groups attained peak HR values corresponding to 95% of the age predicted maximum. Thus, taking into account also RER and [La^−^]_b_ peak values, it can be assumed that maximum exercise capacity had in fact been reached in each condition. $\dot{V}$O_2peak_ did not change in all groups (CMIT 47.6 ± 4.2 vs. 48.0 ± 6.5 mL·kg^−1^·min^−1^, CI 95% −2.2 to 2.9, ES 0.08 *trivial*; DHIT 48.8 ± 5.5 vs. 49.0 ± 4.4 mL·kg^−1^·min^−1^, CI 95% −2.3 to 2.8, ES 0.05 *trivial*; CON 48.4 ± 4.4 vs. 48.3 ± 4.0 mL·kg^−1^·min^−1^, CI 95% −2.6 to 2.5, ES 0.02 *trivial*, at PRE and POST, respectively).

**Table 1:** Values of the main cardiovascular, respiratory, and metabolic parameters obtained during the incremental test.

	**CMIT**				**DHIT**				**CON**			
	**PRE**	**POST**	**CI 95%**	**ES**	**PRE**	**POST**	**CI 95%**	**ES**	**PRE**	**POST**	**CI 95%**	**ES**
$\dot{V}$O_2peak_ (L·min^−1^)	3.24 ± 0.33	3.30 ± 0.34	−0.2 to 0.1	0.18 *trivial*	3.50 ± 0.39	3.51 ± 0.38	−0.1 to 0.1	0.02 *trivial*	3.37 ± 0.44	3.40 ± 0.42	−0.1 to 0.2	0.07 *trivial*
HR_peak_ (bpm)	173 ± 10	170 ± 10	−0.1 to 6.6	0.30 *small*	175 ± 14	172 ± 13	−0.8 to 6.5	0.21 *small*	174 ± 9	173 ± 11	−0.5 to 5.8	0.11 *trivial*
$\dot{V}$E (L·min^−1^)	116.5 ± 12.8	112.9 ± 16.2	−4.0 to 11.2	0.28 *small*	121.1 ± 17.7	129.5 ± 24.6	−12.9 to 17.4	0.47 *small*	119.8 ± 14.2	118.1 ± 15.4	−9.8 to 17.4	0.12 *trivial*
RER	1.19 ± 0.04	1.20 ± 0.07	−0.1 to 0.1	0.25 *small*	1.16 ± 0.07	1.18 ± 0.08	−0.1 to 0.1	0.28 *small*	1.18 ± 0.02	1.19 ± 0.06	−0.1 to 0.1	0.50 *small*
[La^−^]_b_ (mM)	7.81 ± 1.57	8.01 ± 1.64	−1.8 to 0.3	0.12 *trivial*	7.9 ± 0.8	8.44 ± 0.5	−2.5 to 0.1	0.67 *moderate*	8.4 ± 1.1	8.2 ± 0.9	−1.5 to 0.2	0.18 *trivial*

**Figure 1:**
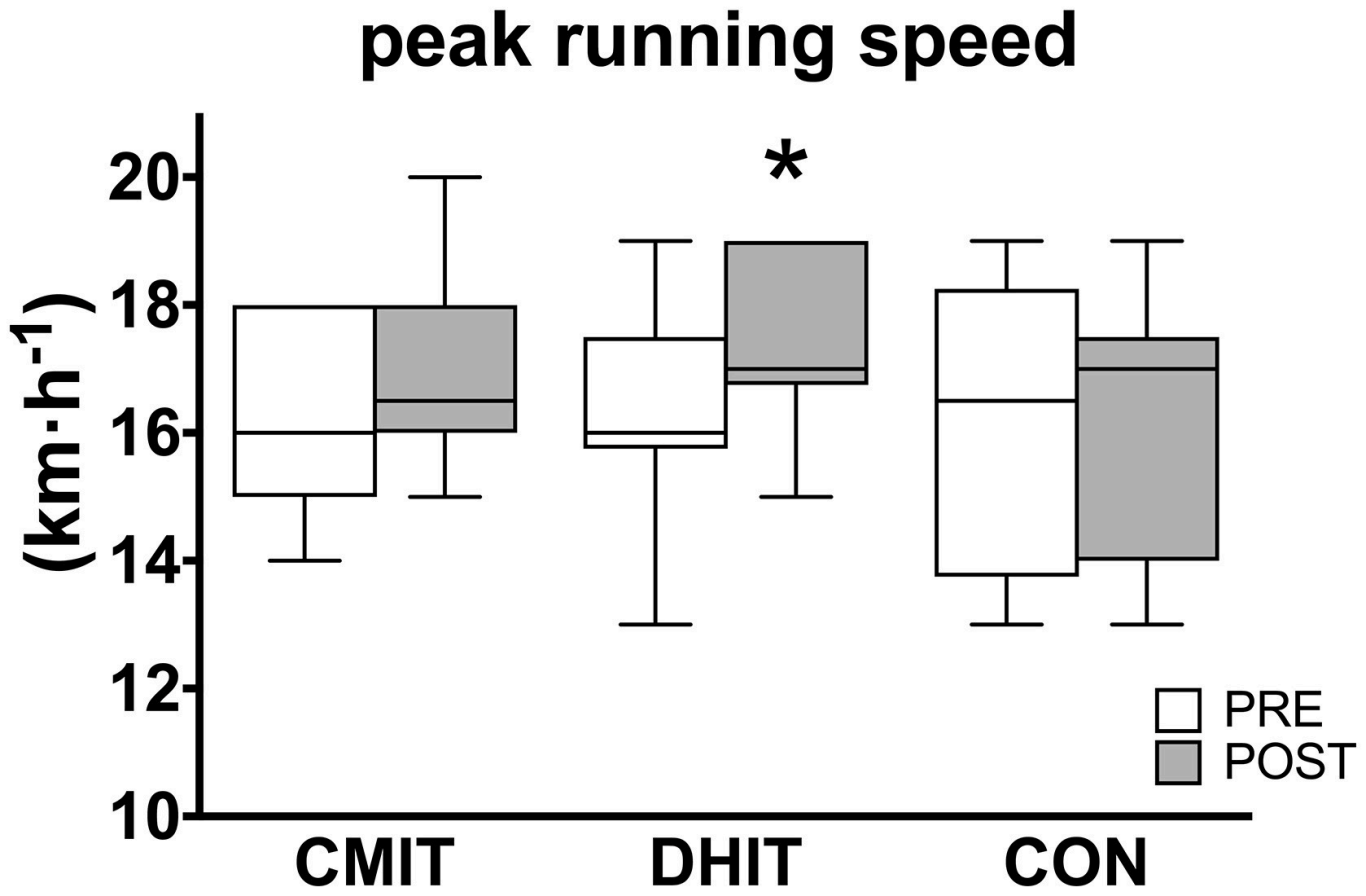
Peak running speed obtained during incremental test before (PRE, white boxes) and after (POST, gray boxes) training intervention. CMIT, continuous moderate intensity training; DHIT, discontinuous high-intensity training; CON, control group. ^*^Significantly different from PRE, *P* < 0.05. The box extends from the 25th percentile to the 75th percentile, with a bold line at the median value.

**Figure 2:**
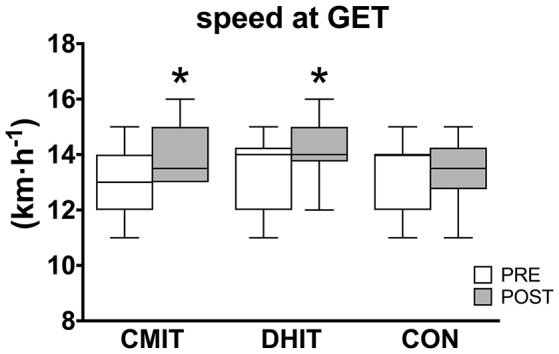
Running speed at gas exchange threshold (GET) before (PRE, white boxes) and after (POST, gray boxes) training intervention. CMIT, continuous moderate intensity training; DHIT, discontinuous high-intensity training; CON, control group. ^*^Significantly different from PRE, *P* < 0.05. The box extends from the 25th percentile to the 75th percentile, with a bold line at the median value.

*Values are means ± SD. CI, confidence interval; ES, effect size; $\dot{V}$O_2peak_, peak oxygen uptake; HR_peak_, peak heart rate; $\dot{V}$E, pulmonary ventilation; [La^−^]_b_, blood lactate concentration; RER, respiratory exchange ratio*.

v_peak_ was significantly higher in POST only in DHIT (16.4 ± 1.8 vs. 17.4 ± 1.3 km·h^−1^, CI 95% 0.3 to 1.7, ES 0.62 *moderate*, at PRE and POST, respectively; *P* = 0.015) whereas no differences were found for CMIT (16.4 ± 1.5 vs. 16.9 ± 1.4 km·h^−1^, CI 95% −0.2 to 1.2, ES 0.31 *small*, at PRE and POST, respectively) and CON (16.3 ± 1.9 vs. 16.5 ± 1.6 km·h^−1^, CI 95% −0.5 to 0.9, ES 0.12 *trivial*, at PRE and POST, respectively; Figure 1). As for GET expressed as percentage of $\dot{V}$O_2peak_, no significant differences were found at POST compared to PRE in CMIT (88.6 ± 3.2 vs. 87.6 ± 6.1%, CI 95% −3.9 to 5.7, ES 0.17 *trivial*), in DHIT (88.8 ± 4.6 vs. 86.8 ± 4.9%, CI 95% −3.6 to 6.0, ES 0.31 *small*) and CON (88.8 ± 3.8 vs. 87.6 ± 6.3%, CI 95% −2.5 to 7.1, ES 0.22 *small*). However, the speed at GET significantly improved in CMIT (14.6 ± 1.4 vs. 15.1 ± 1.1 km·h^−1^, CI 95% 0.2 to 1.4, ES 0.39 *small, P* = 0.023, PRE and POST, respectively) and DHIT (14.1 ± 1.2 vs. 14.9 ± 1.1 km·h^−1^, CI 95% 0.3 to 1.4, ES 0.51 *small, P* = 0.025, PRE and POST, respectively) whereas no differences were found in CON (14.0 ± 1.6 vs. 14.2 ± 1.3 km·h^−1^, CI 95% −0.5 to 0.7, ES 0.15 *trivial*, PRE and POST, respectively; Figure 2). No differences were found among groups.

### Constant load exercise

**Figure 3:**
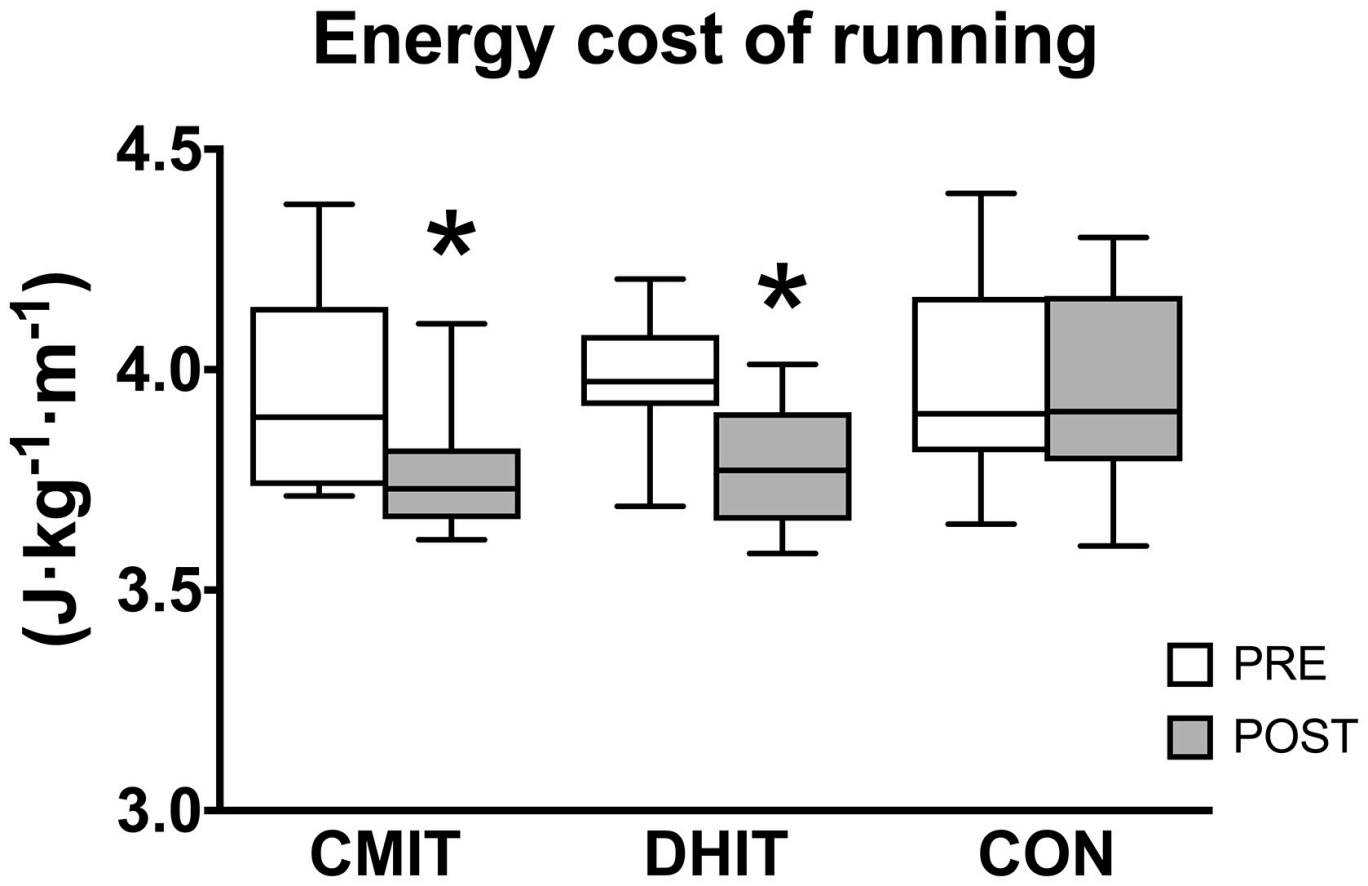
Energy cost of running (Cr) calculated during constant load exercise before (PRE, white boxes) and after (POST, gray boxes) training intervention. CMIT, continuous moderate intensity training; DHIT, discontinuous high-intensity training; CON, control group. ^*^Significantly different from PRE, *P* < 0.05. The box extends from the 25th percentile to the 75th percentile, with a bold line at the median value.

The absolute intensity of CLE did not change for the three groups after training (~10 km·h^−1^). Mean $\dot{V}$E, $\dot{V}$O_2_, RER and, HR values obtained during steady state exercise and Δ[La^−^]_b_ (highest value into recovery—resting value) are presented in Table 2. All variables were significantly (*P* < 0.05) lower POST compared to PRE both in CMIT and DHIT, whereas no changes were observed in CON. Energy cost of running is shown in Figure 3. After training, CMIT and DHIT significantly decreased Cr by 4.4 and 4.9%, respectively. No change was found for CON.

**Table 2:** Values of the main cardiovascular, respiratory, and metabolic parameters obtained during the constant load exercise.

	**CMIT**				**DHIT**				**CON**			
	**PRE**	**POST**	**CI 95%**	**ES**	**PRE**	**POST**	**CI 95%**	**ES**	**PRE**	**POST**	**CI 95%**	**ES**
$\dot{V}$O_2_ss (mL·Kg^−1^·min^−1^)	33.9 ± 2.8	32.6 ± 2.2^*^	1.7 to 5.3	0.46 *small*	33.1 ± 3.3	31.7 ± 3.1^*^	0.8 to 6.1	0.42 *small*	33.3 ± 2.3	33.0 ± 2.1	−1.4 to 3.1	0.13 *trivial*
HR (bpm)	137 ± 12	132 ± 13^*^	1.1 to 10.4	0.41 *small*	129 ± 11	125 ± 10^*^	0.2 to 6.9	0.36 *small*	136 ± 13	134 ± 14	−2.2 to 4.6	0.15 *trivial*
$\dot{V}$E (L·min^−1^)	61.2 ± 9.4	57.1 ± 10.2^*^	3.8 to 13.6	0.43 *small*	60.1 ± 8.2	57.7 ± 6.3^*^	0.3 to 13.1	0.29 *small*	59.7 ± 8.3	58.1 ± 9.0	−0.3 to 3.4	0.19 *trivial*
RER	0.92 ± 0.05	0.89 ± 0.04^*^	0.0 to 0.1	0.6 *moderate*	0.94 ± 0.02	0.91 ± 0.03^*^	0.0 to 0.2	1.50 *large*	0.92 ± 0.02	0.91 ± 0.04	−0.1 to 0.1	0.50 *small*
Δ[La^−^]_b_ (mM)	0.7 ± 0.4	0.4 ± 0.3^*^	0.1 to 0.5	0.75 *moderate*	0.7 ± 0.4	0.3 ± 0.3^*^	0.0 to 0.6	1,00 *moderate*	0.7 ± 0.5	0.5 ± 0.5	−0.1 to 0.3	0.40 *small*

*Values are means ± SD. CI, confidence interval; ES, effect size; $\dot{V}$O_2_ss, oxygen consumption at steady state; HR, heart rate; $\dot{V}$E, pulmonary ventilation; RER, respiratory exchange ratio; Δ[La^−^]_b_, highest value into recovery—resting value*,

* *Significantly different from PRE (P < 0.05)*.

### Running performance

**Figure 4:**
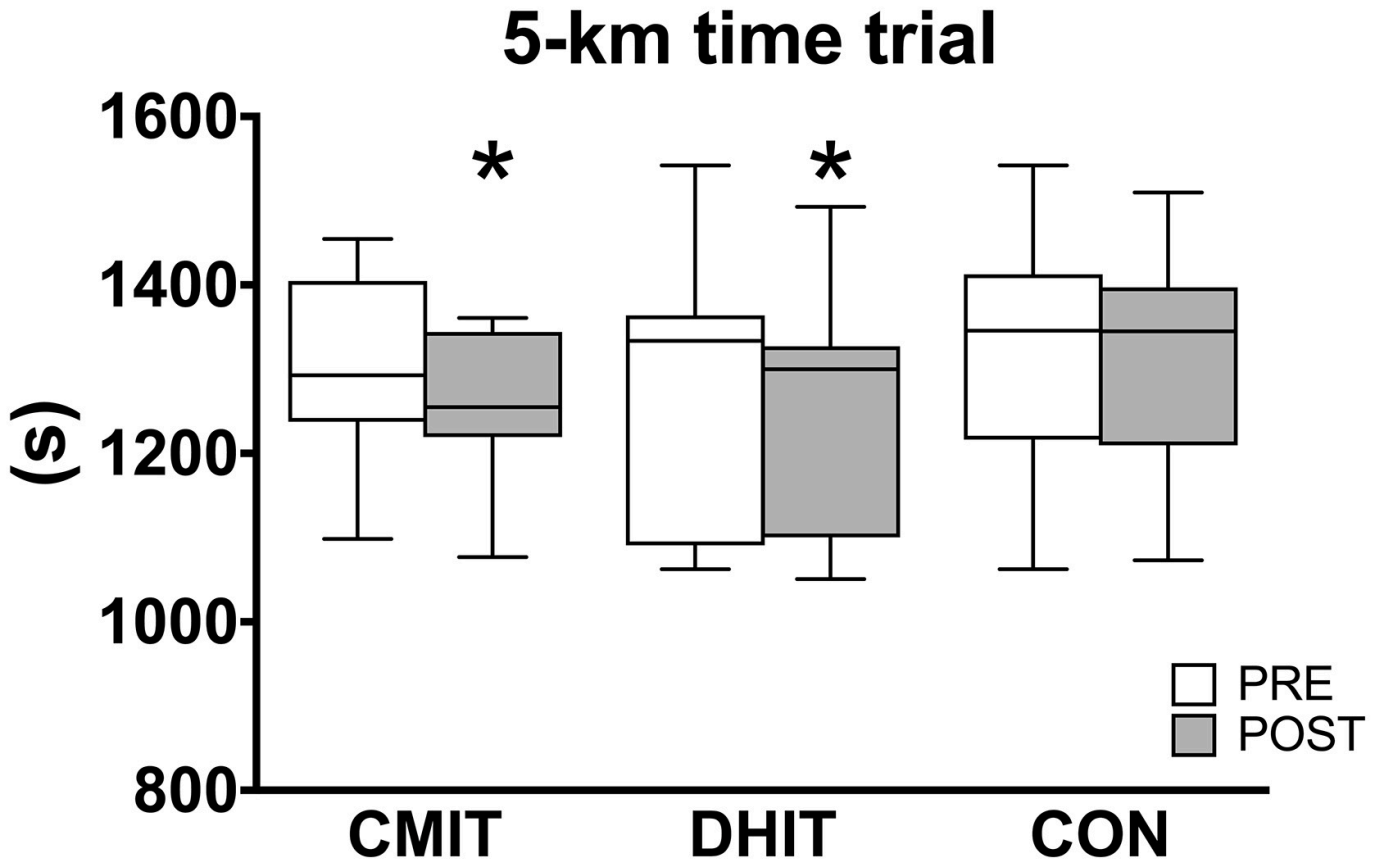
Five kilometers time trial performance on a 400-m outdoor track before (PRE, white boxes) and after (POST, gray boxes) training intervention. CMIT, continuous moderate intensity training; DHIT, discontinuous high-intensity training; CON, control group. ^*^Significantly different from PRE, *P* < 0.05. The box extends from the 25th percentile to the 75th percentile, with a bold line at the median value.

5-km time trial results are shown in Figure 4. At PRE, no significant difference among groups was observed. At POST (vs. PRE), the time to cover 5 km was statistically lower both in CMIT (1264 ± 85 vs. 1304 ± 109 s, *P* = 0.003) and DHIT (1254 ± 140 vs. 1282 ± 155 s, *P* = 0.016). Performance did not change in CON (1309 ± 142 vs. 1320 ± 149 s). Results of the Pearson product moment-correlation analysis showed that all variables measured were significantly correlated with 5-km time trial (Table 3). The stepwise multiple regression analysis performed in PRE on all runners (*n* = 30) revealed that v_peak_ (km·h^−1^), GET (mL·kg^−1^·min^−1^), and Cr (J·kg^−1^·m^−1^) were the variables to be selected for prediction of the 5-km time trial. The analysis showed that 72.4% of the variance in 5-km time trial could be explained by v_peak_ alone (*P* < 0.0001), and the addiction of GET and Cr to the prediction equation increased this significantly to 79.0% (v_peak_ and GET; *P* < 0.0001) and 85.5% (v_peak_, GET and Cr; *P* < 0.0001). Table 4 summarizes the linear multiple regression equations obtained. No differences were found among groups.

**Table 3:** Relationships between $\dot{V}$O_2_peak, Vpeak, GET, Speed at GET, Cr, and 5-km time trial (*n* = 30).

**Variables**	**$\dot{V}$O_2peak_ (mL·kg^−1^·min^−1^)**	**v_peak_ (km·h^−1^)**	**GET (mL·kg-1·min-1)**	**Speed at GET (km·h^−1^)**	**Cr (J·kg^−1^·min^−1^)**
5-km time trial (s)	−0.733^**^	−0.856^**^	−0.695^**^	−0.831^**^	0.428^*^

*$\dot{V}$O_2peak_, peak oxygen uptake; v_peak_, maximal speed at the end of incremental test; GET, gas exchange threshold; Cr, energetic cost*.

** *P < 0.01*,

* *P < 0.05*.

**Table 4:** Linear multiple regression equations (*n* = 30).

5-km time trial (s) = 2353.979–64.350 v_peak_
5-km time trial (s) = 2690.008–51.091 v_peak_-13.166 GET
5-km time trial (s) = 2181.979–26.394 v_peak_-23.977 GET + 146.629 Cr

*v_peak_, maximal speed at the end of incremental test; GET, gas exchange threshold; Cr, energetic cost*.

## Discussion

The main finding of this study was that both moderate-intensity continuous training and high-intensity discontinuous training improve running performance in middle-aged master runners.

Form a physiological point of view, it is widely accepted that running performance depends on maximal oxygen uptake ($\dot{V}$O_2max_), the ability to sustain a high percentage of $\dot{V}$O_2max_ for an extended period of time (*f* $\dot{V}$O_2_), and the energy cost of running (Cr): v = *f* $\dot{V}$O_2max_ · Cr^−1^ (di Prampero, 1986; Capelli, 1999). After training, $\dot{V}$O_2peak_ was unchanged in the master runners participating to the present study. At the same time, in the master runners participating to the present study we have observed a significant decrease of Cr following both CMIT and DHIT (4.4 and 4.9%, respectively). The magnitude of this improvement was close to the change in energy cost of running reported in young trained distance runners after a period of interval training (Franch et al., 1998; Billat et al., 1999; Helgerud et al., 2007). We are unaware of similar studies performed in middle-aged master endurance athletes. Of interest is the observation that the positive effect on Cr was achieved despite a significant reduction of the previous self-managed training volume. Our results are in line with the theoretical model of the performance determinants: v = *f* $\dot{V}$O_2max_·Cr^−1^ (di Prampero, 1986; Capelli, 1999). After training $\dot{V}$O_2peak_, GET and calculated *f* $\dot{V}$O_2max_ (91 and 90% at PRE and POST, respectively) were unchanged. However, the analysis of 5-km finish time with the theoretical model of performance by considering the Cr value in POST lead to a reduction of 3.1% in CMIT, the same as with Capelli's model 1999 and of 2.2% in DHIT, that was lower than the model prediction of 4.9%. Thus, other factors (i.e., biomechanical, neurophysiological, psychological etc.) counterbalanced the reduction of Cr and negatively influenced running performance of DHIT runners.

The energy cost of running, as is well known, is influenced by several metabolic, neuromuscular, and/or biomechanical factors (Saunders et al., 2004; Lacour and Bourdin, 2015). It has been suggested that training interventions able to reduce $\dot{V}$E, HR, and Δ[La^−^]_b_ during exercise may be beneficial to Cr (Saunders et al., 2004). In our study, we observed after training, a significant reduction of $\dot{V}$E and HR at the same running speed both in CMIT and DHIT. However, it is hard to think that these changes were responsible for the overall $\dot{V}$O_2_ reduction and consequently for the Cr decrease. Taking into account the ratio of 1.5–2.0 mL·min^−1^ $\dot{V}$O_2_ per L·min^−1^ of hyperpnea calculated by Aaron et al. (1992), a 2–4 L·min^−1^ difference in $\dot{V}$E should correspond to a reduction of about 3–8 mL O_2_·min^−1^ (0.04–0.11 mL O_2_·min^−1^ when expressed per kilogram body weight). Likewise, assuming a myocardial $\dot{V}$O_2_ of 0.4 mL O_2_ per 100 g of left ventricular mass and per beat (Sheffield, 1988), the reduction of 4–5 b·min^−1^ observed after training should induce a negligible effect on the overall energy balance. Finally, in both PRE and POST the Δ[La^−^]_b_ measured indicates that the metabolic energy produced via anaerobic glycolysis was negligible, as expected for an exercise of moderate intensity, and we can assume that lactate did not contribute significantly to total energy expenditure. Thus, the metabolic factors cannot completely explain the improved energy cost of running.

Beside physiological aspects, neuromuscular, and biomechanical factors may also be important determinants of Cr (Lacour and Bourdin, 2015). Previous studies have reported an improvement of neuromuscular characteristics following plyometric (Spurrs et al., 2003) and strength (Paavolainen et al., 1999) training in young runners. Similar results were observed also in master runners following concurrent strength and endurance training (Piacentini et al., 2013). Moreover, high-intensity training may lead to a high engagement of the neuromuscular system (Buchheit and Laursen, 2013) and improve muscular function, especially in older athletes that experience a decrease in muscle mass and neural function with aging. In our study, we did not directly evaluate neuromuscular function but the improvement in v_peak_ (with no change in $\dot{V}$O_2peak_) for DHIT group after training might be related, at least in part, to improvements in muscular function (Esfarjani and Laursen, 2007). Thus, the reduction of the energy cost of running observed may be due to an improved neuromuscular function, at least in the subjects enrolled in the DHIT group.

From a biomechanical point of view, several parameters can be optimized (e.g., stride frequency, spring mass model related stiffness) or minimized (e.g., mechanical work) in order to improve Cr (Dalleau et al., 1998; Lieberman et al., 2015; Zamparo et al., 2016). However, published data on masters athletes or aged runners seem to show an already optimized system. Cavagna et al. (2008) reported that the freely chosen stride frequency is higher in old runners compared with young, but it is closer to the natural frequency of the system, and with a lower total mechanical work. Pantoja et al. (2016) showed that both vertical and leg stiffness are similar in young and master runners even if Cr is higher in master. Biomechanical analysis was beyond the aims of this study and we did not measure any of the aforementioned parameters, thus we cannot say if any change occurred after training and if they could have affected Cr.

Another finding of this study is that CMIT and DHIT group significantly improved speed at GET by 3.7 and 4.8% for CMIT and DHIT, respectively. It is known that a rightward shift of the GET to a higher running speed is a clear sign of successful endurance training programs. This adaptation allows a higher absolute exercise intensity to be sustained without the accumulation of blood lactate that can be translated in a higher average running speed during competitions (Jones and Carter, 2000). Indeed, participants in both CMIT and DHIT group were able to run the 5-km time trial at higher speed after training (~2.5% faster). Nevertheless, we did not find any change in GET when expressed as a percentage $\dot{V}$O_2peak_. This may be due to an already high pre-training GET value (overall mean ~87% of $\dot{V}$O_2peak_) that is a common feature of endurance master athletes (Kusy et al., 2012), which signified a limited potential for improvements with training.

Previous studies have consistently reported that both CMIT and DHIT induce large improvements in the $\dot{V}$O_2_max of untrained/moderately trained healthy adults aged 18–45 years (for a review see Milanović et al., 2015). In our master runners, however, we did not find any significant change in $\dot{V}$O_2peak_ following the two training programs. When considering this discrepancy, two factors should be taken into account: (1) participants were master runners with several years of training experience; (2) both CMIT and DHIT induced a significant reduction of the previous self-managed training volume that could negatively affected $\dot{V}$O_2peak_. Thus, our data should be observed with a different point of view: a significant reduction in training volume did not change $\dot{V}$O_2peak_ after both CMIT and DHIT whereas the 5-km running performance improved after both training protocols. These findings are interesting because they indicate that when master athletes have reached a good maximal aerobic capacity, other factors could be important in order to obtain further improvements in running performance. This seems to be supported by the stepwise regression analysis performed before the training intervention. Our data showed that all variables (physiological and mechanical) measured were significantly correlated with 5-km performance (Table 3). However, the v_peak_ and speed at GET had the higher correlation coefficient (*r* = −0.856 and *r* = −0.831, respectively). This result supports previous studies, in which v_peak_ and the fractional utilization of the $\dot{V}$O_2peak_ (expressed as lactate or ventilatory threshold) were highly correlated with performance in 5-km in young (Lacour et al., 1990) and middle-aged runners (Takeshima and Tanaka, 1995). Moreover, the stepwise regression analysis revealed that only v_peak_, GET, and Cr predicted the 5-km performance (adjusted *r*^2^ = 0.855). In the present study, DHIT improved Cr, speed at GET and v_peak_ whereas CMIT only influenced Cr and speed at GET. These findings are in accordance with other studies showing that v_peak_, lactate or ventilatory threshold and energy cost may be better predictors of endurance performance than $\dot{V}$O_2peak_ in a homogeneous group of trained endurance athletes (Conley and Krahenbuhl, 1980; Allen et al., 1985; Stratton et al., 2009).

From a practical point of view, it should be highlighted that a sizeable reduction of self-managed training volume replaced by a controlled training program significantly improved performance. Before the intervention the whole group of runners reported to undertake a combination of both high-intensity (~4–10% of total) and high-volume (~ 90% of total) training sessions for a training volume of about 50 km·wk^−1^. However, subjects did not follow any individualized training program and training intensity, as well as training volume was based only on the participants' experience. Previous studies have emphasized that the reduction in the exercise training stimulus with advancing age may have a critical role in the decline of peak performance (Tanaka et al., 1997). Our findings suggest that the quality of training should also be taken into account to improve running performance.

Finally, the master athlete is more likely to experience sports-related injuries because the tissues that make up the tendons, ligaments, cartilage, and muscle break down more easily and heal with greater difficulty (Maharam et al., 1999). Although it was not the aim of this study, we observed a low incidence (one runner in DHIT and one runner in CMIT) of muscular or orthopedic injuries after training intervention. These results indicate that a controlled DHIT program can be safely carried out not only in young athletes (Gibala et al., 2006) but also in master runners as outlined by the observation that, in the same cohort of athletes, DHIT does not cause higher level of exercise-induced oxidative stress than CMIT (Vezzoli et al., 2014).

In conclusion, this study indicates that both well-controlled CMIT and DHIT training program may significantly reduce the amount of weekly training volume (with a consistent reduction of time) and significantly improve running performance in middle-aged master runners, mainly lowering the energy cost of running. v_peak_, fractional utilization of $\dot{V}$O_2peak_, and energy cost appear to be the best predictors of 5-km performance in middle-aged master runners suggesting that training sessions should focus on the improvement of these parameters.

## Author contributions

LP, SP, AL, FS, and MM: participated in the study design; LP, SP, and MM: conducted the experiments; MM: was responsible for medical expertise and screening of the athletes; LP, SP, AV, and GP: performed the data analysis; LP, SP, AV, AL, FS, GP, and MM: wrote or contributed to the writing of the manuscript.

### Conflict of interest statement

The authors declare that the research was conducted in the absence of any commercial or financial relationships that could be construed as a potential conflict of interest.

## Footnotes

^1^Available online at http://www.runningusa.org/marathon-report-2016?returnTo=annual-reports.

## Abbreviations

$\dot{V}$O_2_: oxygen consumption
$\dot{V}$E: pulmonary ventilation
$\dot{V}$CO_2_: Carbon dioxide production
$\dot{V}$O_2peak_: peak oxygen consumption
$\dot{V}$O_2max_: maximal oxygen consumption
*f*$\dot{V}$O_2max_: fraction of maximal oxygen consumption
v_peak_: maximal speed
[La^−^]_b_: blood lactate concentration
BTPS: body temperature, pressure, and saturated
CLE: Constant load exercise
CMIT: continuous training at low- to moderate-intensity
CON: control group
Cr: energy cost of running
DHIT: discontinuous high-intensity training
ECG: electrocardiogram
GET: gas exchange threshold
HR: heart rate
IE: incremental exercise
PO_2_: partial pressure of oxygen
PCO_2_: partial pressure of carbon dioxide
PRE: before training
POST: after training
RER: respiratory exchange ratio
STPD: Standard temperature, pressure, and dry.

## References

[B1] Aaron E. A. Johnson B. D. Seow K. C. Dempsey J. A. (1992). Oxygen cost of exercise hyperpnea: measurements. J. Appl. Physiol. 72, 1810–1817. 10.1152/jappl.1992.72.5.1810 1601790

[B2] Allen W. K. Seals D. R. Hurley B. F. Ehsani A. A. Hagberg J. M. (1985). Lactate threshold and distance-running performance in young and older endurance athletes. J. Appl. Physiol. 58, 1281–1284. 10.1152/jappl.1985.58.4.1281 3988681

[B3] Batterham A. M. Hopkins W. G. (2006). Making meaningful inferences about magnitudes. Int. J. Sports Physiol. Perform. 1, 50–57. 10.1123/ijspp.1.1.50 19114737

[B4] Beaver W. L. Wasserman K. Whipp B. J. (1986). A new method for detecting anaerobic threshold by gas exchange. J. Appl. Physiol. 60, 2020–2027. 10.1152/jappl.1986.60.6.2020 3087938

[B5] Billat V. L. Flechet B. Petit B. Muriaux G. Koralsztein J. P. (1999). Interval training at VO2max: effects on aerobic performance and overtraining markers. Med. Sci. Sports Exerc. 31, 156–163. 10.1097/00005768-199901000-00024 9927024

[B6] Brisswalter J. Nosaka K. (2013). Neuromuscular factors associated with decline in long-distance running performance in master athletes. Sports Med. 43, 51–63. 10.1007/s40279-012-0006-9 23315756

[B7] Buchheit M. Laursen P. B. (2013). High-intensity interval training, solutions to the programming puzzle. Part II: anaerobic energy, neuromuscular load and practical applications. Sports Med. 43, 927–954. 10.1007/s40279-013-0066-5 23832851

[B8] Capelli C. (1999). Physiological determinants of best performances in human locomotion. Eur. J. Appl. Physiol. Occup. Physiol. 80, 298–307. 10.1007/s004210050596 10483799

[B9] Cavagna G. A. Legramandi M. A. Peyré-Tartaruga L. A. (2008). Old men running: mechanical work and elastic bounce. Proc. R. Soc. B 275, 411–418. 10.1098/rspb.2007.1288 18077249 PMC2596824

[B10] Conley D. L. Krahenbuhl G. S. (1980). Running economy and distance running performance of highly trained athletes. Med. Sci. Sports Exerc. 12, 357–360. 10.1249/00005768-198025000-00010 7453514

[B11] Dalleau G. Belli A. Bourdin M. Lacour J. R. (1998). The spring-mass model and the energy cost of treadmill running. Eur. J. Appl. Physiol. 77, 257–263. 10.1007/s004210050330 9535587

[B12] di Prampero P. E. (1986). The energy cost of human locomotion on land and in water. Int. J. Sports Med. 7, 55–72. 10.1055/s-2008-1025736 3519480

[B13] Donato A. J. Tench K. Glueck D. H. Seals D. R. Eskurza I. Tanaka H. (2003). Declines in physiological functional capacity with age: a longitudinal study in peak swimming performance. J. Appl. Physiol. 94, 764–769. 10.1152/japplphysiol.00438.2002 12391125 PMC5063028

[B14] Esfarjani F. Laursen P. B. (2007). Manipulating high-intensity interval training: effects on VO2max, the lactate threshold and 3000 m running performance in moderately trained males. J. Sci. Med. Sport 10, 27–35. 10.1016/j.jsams.2006.05.014 16876479

[B15] Franch J. Madsen K. Djurhuus M. S. Pedersen P. K. (1998). Improved running economy following intensified training correlates with reduced ventilatory demands. Med. Sci. Sports Exerc. 30, 1250–1256. 10.1097/00005768-199808000-00011 9710865

[B16] Gibala M. J. Little J. P. van Essen M. Wilkin G. P. Burgomaster K. A. Safdar A.. (2006). Short-term sprint interval versus traditional endurance training: similar initial adaptations in human skeletal muscle and exercise performance. J. Physiol. 575, 901–911. 10.1113/jphysiol.2006.112094 16825308 PMC1995688

[B17] Helgerud J. Høydal K. Wang E. Karlsen T. Berg P. Bjerkaas M.. (2007). Aerobic high-intensity intervals improve VO2max more than moderate training. Med. Sci. Sports Exerc. 39, 665–671. 10.1249/mss.0b013e3180304570 17414804

[B18] Holloszy J. O. Coyle E. F. (1984). Adaptations of skeletal muscle to endurance exercise and their metabolic consequences. J. Appl. Physiol. Respir. Environ. Exerc. Physiol. 56, 831–838. 10.1152/jappl.1984.56.4.831 6373687

[B19] Iaia F. M. Hellsten Y. Nielsen J. J. Fernström M. Sahlin K. Bangsbo J. (2009). Four weeks of speed endurance training reduces energy expenditure during exercise and maintains muscle oxidative capacity despite a reduction in training volume. J. Appl. Physiol. 106, 73–80. 10.1152/japplphysiol.90676.2008 18845781

[B20] Jones A. M. Carter H. (2000). The effect of endurance training on parameters of aerobic fitness. Sports Med. 29, 373–386. 10.2165/00007256-200029060-00001 10870864

[B21] Jones A. M. Doust J. H. (1996). A 1% treadmill grade most accurately reflects the energetic cost of outdoor running. J. Sports Sci. 14, 321–327. 10.1080/02640419608727717 8887211

[B22] Kubukeli Z. N. Noakes T. D. Dennis S. C. (2002). Training techniques to improve endurance exercise performances. Sports Med. 32, 489–509. 10.2165/00007256-200232080-00002 12076176

[B23] Kusy K. Krol-Zielinska M. Domaszewska K. Krysciak J. Podgorski T. Zielinski J. (2012). Gas exchange threshold in male speed-power versus endurance athletes ages 20–90 years. Med. Sci. Sports Exerc. 44, 2415–2422. 10.1249/MSS.0b013e318267c36f 22776883

[B24] Lacour J. R. Bourdin M. (2015). Factors affecting the energy cost of level running at submaximal speed. Eur. J. Appl. Physiol. 115, 651–673. 10.1007/s00421-015-3115-y 25681108

[B25] Lacour J. R. Padilla-Magunacelaya S. Barthélémy J. C. Dormois D. (1990). The energetics of middle-distance running. Eur. J. Appl. Physiol. Occup. Physiol. 60, 38–43. 10.1007/BF00572183 2311592

[B26] Laursen P. B. (2010). Training for intense exercise performance: high-intensity or high-volume training? Scand. J. Med. Sci. Sports 20, 1–10. 10.1111/j.1600-0838.2010.01184.x 20840557

[B27] Laursen P. B. Jenkins D. G. (2002). The scientific basis for high-intensity interval training: optimising training programmes and maximising performance in highly trained endurance athletes. Sports Med. 32, 53–73. 10.2165/00007256-200232010-00003 11772161

[B28] Lieberman D. E. Warrener A. G. Wang J. Castillo E. R. (2015). Effects of stride frequency and foot position at landing on braking force, hip torque, impact peak force and the metabolic cost of running in humans. J. Exp. Biol. 218, 3406–3414. 10.1242/jeb.125500 26538175

[B29] Maharam L. G. Bauman P. A. Kalman D. Skolnik H. Perle S. M. (1999). Master athletes: factor affecting performance. Sports Med. 28, 273–285. 10.2165/00007256-199928040-00005 10565553

[B30] Margaria R. Cerretelli P. Aghemo P. Sassi G. (1963). Energy cost of running. J. Appl. Physiol. 18, 367–370. 10.1152/jappl.1963.18.2.367 13932993

[B31] Milanović Z. Sporiš G. Weston M. (2015). Effectiveness of high-intensity interval training (HIT) and continuous endurance training for VO2max improvements: a systematic review and meta-analysis of controlled trials. Sports Med. 45, 1469–1481. 10.1007/s40279-015-0365-0 26243014

[B32] Paavolainen L. Häkkinen K. Hämälainen I. Nummela A. Rusko H. (1999). Explosive-strength training improves 5-km running time by improving running economy and muscle power. J. Appl. Physiol. 86, 1527–1533. 10.1152/jappl.1999.86.5.1527 10233114

[B33] Pantoja P. D. Morin J. B. Peyré-Tartaruga L. A. Brisswalter J. (2016). Running energy cost and spring-mass behavior in young versus older trained athletes. Med. Sci. Sports Exerc. 9, 1779–1786. 10.1249/MSS.0000000000000959 27116643

[B34] Piacentini M. F. De Ioannon G. Comotto S. Spedicato A. Vernillo G. La Torre A. (2013). Concurrent strength and endurance training effects on running economy in master endurance runners. J. Strength Cond. Res. 27, 2295–2303. 10.1519/JSC.0b013e3182794485 23207882

[B35] Reaburn P. Dascombe B. (2008). Endurance performance in masters athletes. Eur. Rev. Aging Phys. Act. 5, 31–42. 10.1007/s11556-008-0029-2

[B36] Saunders P. U. Pyne D. B. Telford R. D. Hawley J. A. (2004). Factors affecting running economy in trained distance runners. Sports Med. 34, 465–485. 10.2165/00007256-200434070-00005 15233599

[B37] Seiler K. S. Kjerland G. (2006). Quantifying training intensity distribution in elite endurance athletes: is there evidence for an “optimal” distribution? Scand. J. Med. Sci. Sports 16, 49–56. 10.1111/j.1600-0838.2004.00418.x 16430681

[B38] Sheffield L. T. (1988). Exercise stress testing, in Heart Disease: A Text Book of Cardiovascular Medicine, ed Braunwald E. (Philadelphia, PA: W. B. Saunders), 224.

[B39] Spurrs R. W. Murphy A. J. Watsford A. J. (2003). The effect of plyometric training on distance running performance. Eur. J. Appl. Physiol. 89, 1–7. 10.1007/s00421-002-0741-y 12627298

[B40] Stratton E. O'Brien B. J. Harvey J. Blitvich J. McNicol A. J. Janissen D.. (2009). Treadmill velocity best predicts 5000-m run performance. Int. J. Sports Med. 30, 40–45. 10.1055/s-2008-1038761 19202577

[B41] Takeshima N. Tanaka K. (1995). Prediction of endurance running performance for middle-aged and older runners. Br. J. Sports Med. 29, 20–23. 10.1136/bjsm.29.1.20 7788211 PMC1332212

[B42] Tanaka H. Desouza C. A. Jones P. P. Stevenson E. T. Davy K. P. Seals D. R. (1997). Greater rate of decline in maximal aerobic capacity with age in physically active vs. sedentary healthy women. J. Appl. Physiol. 83, 1947–1953. 10.1152/jappl.1997.83.6.1947 9390967

[B43] Tanaka H. Seals D. R. (2008). Endurance exercise performance in masters athletes: age-associated changes and underlying physiological mechanisms. J. Physiol. 586, 55–63. 10.1113/jphysiol.2007.141879 17717011 PMC2375571

[B44] Trappe S. Hayes E. Galpin A. Kaminsky L. Jemiolo B. Fink W.. (2013). New records in aerobic power among octogenarian lifelong endurance athletes. J. Appl. Physiol. 114, 3–10. 10.1152/japplphysiol.01107.2012 23065759 PMC3544519

[B45] Vezzoli A. Pugliese L. Marzorati M. Serpiello F. R. La Torre A. Porcelli S. (2014). Time-course changes of oxidative stress response to high-intensity discontinuous training versus moderate-intensity continuous training in masters runners. PLoS ONE 9:e87506. 10.1371/journal.pone.0087506 24498121 PMC3909150

[B46] Zamparo P. Pavei G. Nardello F. Bartolini D. Monte A. Minetti A. E. (2016). Mechanical work and efficiency of 5 + 5 m shuttle running. Eur. J. Appl. Physiol. 116, 1911–1919. 10.1007/s00421-016-3443-6 27473448

